# The Neurogenic Effects of Exogenous Neuropeptide Y: Early Molecular Events and Long-Lasting Effects in the Hippocampus of Trimethyltin-Treated Rats

**DOI:** 10.1371/journal.pone.0088294

**Published:** 2014-02-07

**Authors:** Valentina Corvino, Elisa Marchese, Maria Vittoria Podda, Wanda Lattanzi, Stefano Giannetti, Valentina Di Maria, Sara Cocco, Claudio Grassi, Fabrizio Michetti, Maria Concetta Geloso

**Affiliations:** 1 Institute of Anatomy and Cell Biology - Università Cattolica del Sacro Cuore, Rome, Italy; 2 Institute of Human Physiology - Università Cattolica del Sacro Cuore, Rome, Italy; Oregon Health & Science University, United States of America

## Abstract

Modulation of endogenous neurogenesis is regarded as a promising challenge in neuroprotection. In the rat model of hippocampal neurodegeneration obtained by Trimethyltin (TMT) administration (8 mg/kg), characterised by selective pyramidal cell loss, enhanced neurogenesis, seizures and cognitive impairment, we previously demonstrated a proliferative role of exogenous neuropeptide Y (NPY), on dentate progenitors in the early phases of neurodegeneration. To investigate the functional integration of newly-born neurons, here we studied in adult rats the long-term effects of intracerebroventricular administration of NPY (2 µg/2 µl, 4 days after TMT-treatment), which plays an adjuvant role in neurodegeneration and epilepsy. Our results indicate that 30 days after NPY administration the number of new neurons was still higher in TMT+NPY-treated rats than in control+saline group. As a functional correlate of the integration of new neurons into the hippocampal network, long-term potentiation recorded in Dentate Gyrus (DG) in the absence of GABA_A_ receptor blockade was higher in the TMT+NPY-treated group than in all other groups. Furthermore, qPCR analysis of Kruppel-like factor 9, a transcription factor essential for late-phase maturation of neurons in the DG, and of the cyclin-dependent kinase 5, critically involved in the maturation and dendrite extension of newly-born neurons, revealed a significant up-regulation of both genes in TMT+NPY-treated rats compared with all other groups. To explore the early molecular events activated by NPY administration, the Sonic Hedgehog (Shh) signalling pathway, which participates in the maintenance of the neurogenic hippocampal niche, was evaluated by qPCR 1, 3 and 5 days after NPY-treatment. An early significant up-regulation of Shh expression was detected in TMT+NPY-treated rats compared with all other groups, associated with a modulation of downstream genes. Our data indicate that the neurogenic effect of NPY administration during TMT-induced neurodegeneration involves early Shh pathway activation and results in a functional integration of newly-generated neurons into the local circuit.

## Introduction

Hippocampal neurogenesis is a highly regulated process which, throughout adult mammalian life, generates new cells differentiating into functionally integrated granular neurons. As this form of brain plasticity can be influenced by endogenous and exogenous factors, as well as by pathological conditions [1]–[3] including neurodegenerative diseases and epilepsy [1], [4], the modulation of adult neurogenesis constitutes an attractive research field. In particular, the pharmacological targeting of endogenous mechanisms may offer clues useful to the development of novel therapeutic strategies in the damaged brain.

In this regard, recent evidence largely supports the involvement of neuropeptide Y (NPY) in the modulation of endogenous neurogenesis [5]–[7]. NPY is a 36 amino acid polypeptide neurotransmitter widely distributed in the mammalian central nervous system (CNS), that exerts a role in various physiological functions, including the regulation of blood pressure, circadian rhythms, feeding behaviour, anxiety, memory processing and cognition [5]–[7]. Within the dentate gyrus (DG), NPY is expressed by a subpopulation of GABAergic interneurons located in the hilus, which innervate the granule cell layer in close proximity to the subgranular zone (SGZ), thus contributing to the physiological control of hippocampal progenitor cells [8]. The pro-neurogenic role of exogenous NPY on hippocampal neurogenic niche has been evidenced both *in vitro* [9]–[11] and *in vivo* [12]. This effect appears to be mediated specifically by the Y1 receptors, and to involve the ERK1/2 signalling pathway [9] and is restricted to nestin, beta tubulin and doublecortin-expressing progenitors [9], [13].

The NPY system is also involved in epilepsy [14], [15], which is known to increase adult neurogenesis acutely [4]. In this respect, a possible anticonvulsant and neuroprotective role has been proposed [14], [16]. Changes in hippocampal NPY levels have been observed in GABAergic interneurons and mossy fibres in both experimental models and human temporal lobe epilepsy (TLE) [14], [15], including the model of hippocampal neurodegeneration and TLE induced by Trimethyltin (TMT) administration [17]–[20].

TMT is an organotin compound with neurotoxicant effects selectively involving the limbic system and especially marked in the hippocampus. Rats exposed to TMT show severe loss of pyramidal neurons in the CA3/hilus and CA1 hippocampal subfields, with selective sparing of GABAergic interneurons expressing parvalbumin and calretinin [17], [18], [21]–[24]. Neuronal damage shows a subacute pattern developing over three weeks [25] and is associated with astroglial and microglial activation [26]–[29], enhanced neurogenesis [30], seizures and cognitive impairment [17], [18]. While the mechanisms by which TMT induces neurodegeneration are still not conclusively clarified, many hypotheses suggest that neuronal damage could be largely dependent on calcium overload, possibly as a consequence of its release from intracellular stores [24], [31], [32].

TMT is thus widely considered a useful instrument to study neuronal and glial factors involved in selective neuronal death, as well as the molecular mechanisms leading to hippocampal neurodegeneration (including neuroinflammation, excitotoxicity, mitochondrial dysfunction and oxidative stress). It also offers a valuable tool to study cell-cell interactions and signalling pathways that modulate injury-induced neurogenesis, including the involvement of newly-generated neurons in the possible repair processes [18], [33].

In this regard, we previously studied the role of exogenous NPY [34] on the hippocampal neurogenic niche of TMT-treated rats. In particular, our previous data indicated a neuroprotective and neurogenic role of exogenous NPY in the early phases of TMT-induced neurodegeneration, mediated by the up-regulation of Bcl-2, Bcl-2l, Bdnf, Sox-2, NeuroD1, Noggin and Doublecortin genes, and accompanied by the early induction of Y1, Y2 and Y5 receptor mRNA [34].

Since the early molecular events triggered by NPY administration and mediating its neurogenic effect have not been definitively clarified, in the present study we explored the possible role of molecular pathways critically involved in the establishment and maintenance of the adult hippocampal neurogenic niche, such as that involving Sonic Hedgehog (Shh) signalling. Shh is known to participate in CNS development and recent evidence highlights its role also in the adult brain [35], where it regulates both cell proliferation [36], [37] and the production of growth and angiogenic factors [38]. In particular it appears to be crucially required for DG progenitor cell proliferation via primary cilia [35], [39], [40], microtubule-based organelles functionally implicated in many developmental processes [41].

The efficacy of adult neurogenesis modulation in achieving structural/functional repair depends on the survival and functional integration of newly-generated neurons into neuronal circuitries. The long-term neurogenic effects of intracerebroventricular (i.c.v.) NPY administration were therefore investigated by evaluating morphological, molecular and functional correlates of newly-born neuron integration into the DG network.

## Materials and Methods

### Ethics Statement

All animal procedures were approved by the Ethics Committee of the Catholic University and were fully compliant with The Italian Ministry of Health guidelines (Legislative Decree No. 116/1992) and European Union (Directive No. 86/609/EEC) legislation on animal research. Efforts were made to limit the number of animals used and to minimise their suffering. ARRIVE guidelines were followed.

### Animal Treatment

Adult female Wistar rats (250 g) received a single intra-peritoneal (i.p.) injection of TMT chloride (Sigma, St Louis, MO) dissolved in saline at a dose of 8 mg/Kg body weight in a volume of 1 ml/kg body weight, as previously described [21], [22], [25]; control (CTRL) rats received the same volume of saline. On post-treatment day 4, animals were divided into different experimental groups: CTRL+saline, CTRL+NPY, TMT+saline, TMT+NPY and, under deep anaesthesia, they received i.c.v. administration of NPY (2 µg/2 µl), or the same volume of saline. The animals were anaesthetised with diazepam 2 mg/100 g i.p., followed by ketamine 4 mg/100 g (intramuscular). As previously described [34], they were mounted on a stereotaxic frame; a small parietal hole was made in the skull and a single suspension of NPY (AnaSpec, San Jose, CA, USA) or the same volume of saline, was slowly injected using a Hamilton syringe and the following coordinates: A.P.:+0.80; L.:−1.6; P.:–3.4 from the dura mater [42]. The dose of NPY was chosen according to previous studies [34].

In some experiments CTRL (n = 3) and TMT-treated rats (n = 3) were injected with scrambled NPY peptide (Tocris Biosciences – R&D Systems Company) (4 days after TMT-treatment), at the same dosage as NPY-treated groups (2 µg/2 µl) and sacrificed 1 and 3 days after scrambled NPY treatment.

No body weight variation depending on NPY-administration was observed, as previously described [34].

Animals were returned to their cages and housed on a 12 h light/dark cycle with free access to food and water. One hour after surgical procedures all animals received bromodeoxyuridine (BrdU) (50 mg/kg dissolved in a saline solution 0.1 M NH_4_OH) i.p. BrdU was administered once a day for 5 consecutive days. This schedule was chosen to reduce possible long-term BrdU dilution effects.

### Immunocytochemistry

Rats intended for histology and immunocytochemistry were sacrificed 30 days after NPY or saline i.c.v. administration. Under deep anaesthesia (ketamine/diazepam 1∶1 i.p.), the animals were perfused with 4% phosphate-buffered saline (PBS) paraformaldehyde, the brains were removed from the skull and 40 µm serial sagittal sections were collected in PBS and used for Nissl-staining or immunocytochemistry.

For BrdU labelling, sections were incubated for 30 min with 2N HCl at 37°C for DNA denaturation, for 15 min with 5% normal goat serum at 37°C and overnight with rat monoclonal anti-BrdU antibody (Abcam, Cambridge, UK, 1∶500). The reaction was developed with an avidin-biotin peroxidase complex (ABC method, Vector Burlingame, CA). 3,3′-diaminobenzidine (Sigma, St. Louis, MO) was used as a chromogen.

Mature newly-born neurons were identified by double labelling with the proliferation marker BrdU and the mature neuron marker NeuN (Chemicon, Temecula, CA, 1∶1000, overnight at 4°C) and revealed using donkey anti-mouse cyanine-conjugated secondary antibody (Cy3, 1∶400, 1 h at room temperature, Jackson Immunoresearch Laboratories, West Grove, PA), while BrdU labelling was revealed with FITC conjugated goat anti-rat secondary antibody (Vector, UK, 1∶200, 1 h at room temperature). Controls were prepared by omitting the primary antibody. The co-localisation of the different markers was examined with a Zeiss LSM 510 META confocal laser scanning microscopy system (Zeiss, Oberkochen, Germany).

### Quantitative Analysis

Since it is well known that new granule cells derive from dividing progenitors in the SGZ and migrate approximately 2 cell body widths from the SGZ into the granule layer (GL), where they become functionally integrated into the hippocampal circuit [43], only round, regularly shaped BrdU-positive nuclei located in the DG GL, without signs of nuclear fragmentation, were counted in the four experimental groups (CTRL+saline: n = 7, CTRL+NPY: n = 6, TMT+saline: n = 8, TMT+NPY: n = 7). BrdU-labelled cells with morphological characteristics of glial precursors, i.e. small (diameter <8 µm), irregularly shaped cell bodies, were excluded, as previously described [30].

The optical fractionator stereological method [44] was used to obtain unbiased estimates of total BrdU-positive nuclei in the DG, using the Stereo Investigator system (Stereo Investigator software, Version 9, MicroBrightField Europe, Magdeburg, Germany). A stack of MAC 6000 controller modules (MBF Bioscience, Williston VT, USA) was configured to interface a Nikon Eclipse 80i microscope with a motorised stage and a digital colour camera (MBF Bioscience q imaging) with a Pentium II PC workstation. A three-dimensional optical dissector counting probe (x, y, z dimension of 130 µm × 150 µm × 10 µm respectively) was applied to a systematic random sample of sites in the region of interest at a magnification of 40×. To count BrdU-positive cells in the DG, 1-in-7 series of sagittal sections were taken through the dorsal hippocampus along the septo-temporal axis, from 0.9 to 3.4 mm lateral to the midline, according to Paxinos and Watson’s atlas [42].

BrdU/NeuN double-stained cells in the DG were quantified in the four experimental groups (CTRL+saline: n = 3, CTRL+NPY: n = 3, TMT+saline: n = 3, TMT+NPY: n = 3) using z-scan confocal microscopy at 40X magnification. The entire length of the DG (upper and lower blades) was evaluated through the septo-temporal axis of the hippocampus in 1-in-12 series of sections, as previously described [34], [45]–[47]. Each cell was examined with a multi-channel configuration and only those cells for which the nucleus was unequivocally associated with the neuronal marker were considered as positive [46]. Quantitative analysis was performed as previously described [21], [22], [26], [30], [48]. The number of double-labelled cells was counted manually by an experimenter who was not informed of the group assignment. Estimates of the total number of cells positive for each marker were obtained using the following formula: E = k ΣN, where E is the estimate of the total number of stained cells in each case, ΣN is the sum of n values in the n sections considered, and k indicates that every k^th^ section was considered (k = 12). N was corrected according to Abercrombie’s formula: N = n t/(t+D), where n is the number of cells counted in each section, t is the section thickness, and D is the mean diameter of the cells [49].

The quantification of double-stained cells was expressed as the percentage of double-labelled cells in relation to the total number of BrdU-positive cells or as the total number of double-labelled cells.

### Long-term Potentiation Recordings

Electrophysiological recordings and long-term potentiation (LTP) evaluation at the medial perforant path (MPP)-dentate granule cell (DGC) synapses were performed in slices from rats belonging to the four experimental groups, sacrificed 30–40 days after NPY-treatment. In particular, coronal hippocampal slices (400 µm thick) were obtained according to standard procedures [50]–[52] from 20 rats (CTRL+saline: n = 5, TMT+saline: n = 5, CTRL+NPY: n = 5, TMT+NPY: n = 5). Operators were unaware of the origin of the experimental group being processed.

The rats were anaesthetised with isoflurane and decapitated. The brain was rapidly removed and put in ice-cold cutting solution containing in mM: 124 NaCl, 3.2 KCl, 1 NaH_2_PO_4_, 26 NaHCO_3_, 2 MgCl_2_, 1 CaCl_2_, 10 glucose, 2 Na-pyruvate, and 0.6 ascorbic acid (pH 7.4, 95% O_2_/5% CO_2_). Slices were cut with a vibratome (VT1000S, Leica Microsystems), incubated in the cutting solution at 30–32°C for at least 60 min, and then stored in the same solution at room temperature until use.

For electrophysiological recordings, slices were transferred to a submerged recording chamber and continuously perfused with artificial cerebrospinal fluid (aCSF) containing (in mM): 124 NaCl, 3.2 KCl, 1 NaH_2_PO_4_, 1 MgCl_2_, 2 CaCl_2_, 26 NaHCO_3_, and 10 glucose (pH 7.4, 95% O_2_/5% CO_2_). The flow rate was kept at 1.5 ml/min with a peristaltic pump (Minipuls 3, Gilson, Villiers, France), and bath temperature was maintained at 30–32°C with an in-line solution heater and temperature controller (TC-344B, Warner Instruments, Hamden, CT, USA).

Field excitatory postsynaptic potentials (fEPSPs) were recorded in the hippocampal DG. For this purpose, a stimulating bipolar tungsten electrode (Warner Instruments) connected to a S11 Grass stimulator (Grass Instruments, Quincy, MA) was positioned in the MPP, and a glass capillary microelectrode filled with aCSF (tip resistance 2–5 MΩ) was placed in the middle third of the molecular layer of the DG (upper blade) and connected to a MultiClamp 700A amplifier (Molecular Devices, Sunnyvale, CA, USA). Data acquisition and stimulation protocols were performed with the Digidata 1440 Series interface and pClamp 10 software (Molecular Devices). Data were filtered at 1 kHz, digitised at 10 kHz, and analysed both online and offline.

Hippocampal subfields were identified and electrodes were positioned with the aid of 4X and 40X water immersion objectives on an upright microscope (BX5IWI, Olympus) equipped with a video camera (C3077-71 CCD, Hamamatsu Photonics, Japan).

Isolation of the MPP was confirmed by assessing paired-pulse depression (PPD) (interstimulus interval, 50 ms) at MPP-DGC [53], [54]. The stimulation intensity that produced one-third of the maximal response was used for the test pulses and high frequency stimulation protocol (HFS).

After 10 min of stable baseline responses to test stimulations delivered once every 20 s, LTP was induced with a standard high frequency stimulation (HFS) paradigm consisting of 4 trains of 50 stimuli at 100 Hz (500 ms each) repeated every 20 seconds [53], [55]. Responses to test pulse were recorded every 20 seconds for 30 minutes to assess LTP. The amplitude of fEPSPs at 30 minutes was averaged from values obtained during the last 5 minutes of post-HFS recordings (from minute 25 to minute 30). LTP magnitude was expressed as the percentage change in the mean fEPSP peak amplitude normalised to baseline values (i.e. mean values for the last 10 minutes of recording before HFS, taken as 100%). The fEPSP amplitude was measured from baseline to peak. At the end of LTP recording, all slices included in the study exhibited stable increases of at least 10% in fEPSP amplitude.

### Gene Expression Analysis

Animals intended for quantitative real-time PCR (qPCR) were sacrificed by decapitation after deep anaesthesia (ketamine/diazepam 1∶1 i.p.) 1, 3, 5 and 30 days after NPY, scrambled NPY peptide or saline i.c.v. administration (CTRL+saline: n = 3, CTRL+NPY: n = 3, TMT+saline: n = 3, TMT+NPY: n = 3). The hippocampus homolateral to the injection site was removed and processed for total RNA isolation.

Quantitative real time PCR (qPCR) was used to amplify, in the four experimental groups, the following genes: Sonic hedgehog (Shh), Patched 1 (Ptch1), Cyclin D1 (Ccnd1), Kinesin family member 3a (Kif3a) 1, 3, 5 days following NPY administration, Kruppel-like factor 9 (Klf9) and Cyclin Dependent Kinase 5 (Cdk5) 30 days following NPY administration.

The expression of NPY specific receptors (NpyR) NpyR-1, -2 and -5 was analysed by qPCR in the four experimental groups and in scrambled NPY-treated animals 1 and 3 days after NPY treatment.

Three animals per experimental group were used for the gene expression analysis. The entire qPCR protocol has already been described in detail elsewhere [34]. Briefly, total RNA was isolated from the entire hippocampus of each animal using Trizol reagent (Invitrogen, Carlsbad, CA, USA) and purified using the RNeasy MiniElute Cleanup Kit (Qiagen, Valencia, CA, USA), according to the manufacturer’s instructions, followed by DNAse digestion (Invitrogen). Two-step reverse transcription and qPCR were carried out as previously described [56]. The oligonucleotide primers were designed using the Primer 3 software (http://primer3 sourceforge.net/; primer sequences can be found in Table S1). The 2^−ΔΔCt^ method was applied to calculate the relative quantity (RQ) of gene expression using the housekeeping gene glyceraldehyde-3-phosphate dehydrogenase (Gapdh) for data normalisation [56].

### Statistical Analysis

For statistical analysis, group differences (TMT vs. control and NPY vs. saline) were evaluated using analysis of variance (two-way ANOVA; StatView software); when appropriate, *post hoc* comparisons were made using Bonferroni’s test, with a significance level of p<0.05; data are expressed as mean ± S.E.M.

In order to assess the statistical significance of the gene expression changes for each gene in each experimental group, an unpaired t-test was used to compare the ΔCt values across the replicates, setting the p-value cut-off at 0.05.

Student’s t-test was also used for statistical comparison of data obtained from LTP recordings and data are expressed as mean ± S.E.M.

## Results

### NPY-induced Modulation of Hippocampal Neurogenesis Results in the Functional Integration of Newly-generated Neurons into the Local Circuit in TMT-treated Rats

**Figure 1:**
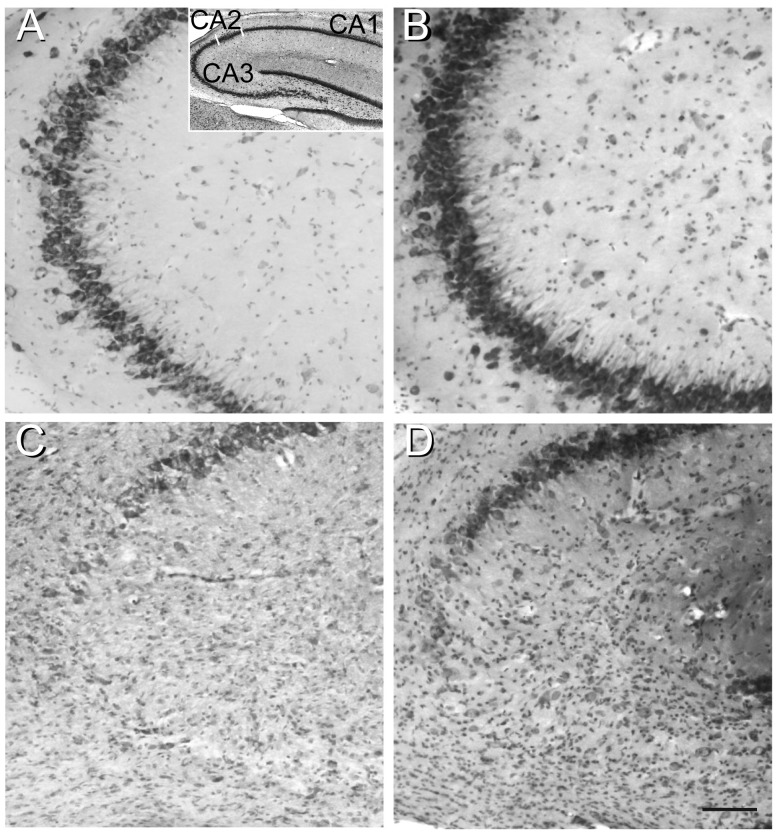
TMT-induced neuronal loss in the rat hippocampus. Micrographs of Nissl-stained 40 µm sagittal sections showing the CA3 subfield, as indicated in the image in the box, from dorsal hippocampus of CTRL+saline- (A), CTRL+NPY- (B), TMT+saline- (C) and TMT+NPY- (D) treated rats. Severe pyramidal neuronal loss is evident in both groups of TMT-treated animals (C, D). Scale bar: 160 µm.

The pattern of TMT-induced neuronal death was consistent with previous observations, as expected [17], [25]. Histological observation of Nissl-stained sections showed neuronal loss mainly localised in the CA3/hilus and CA1 hippocampal subfields of both groups of TMT-treated animals [21], [22], [25] (Fig. 1 A–D).

**Figure 2:**
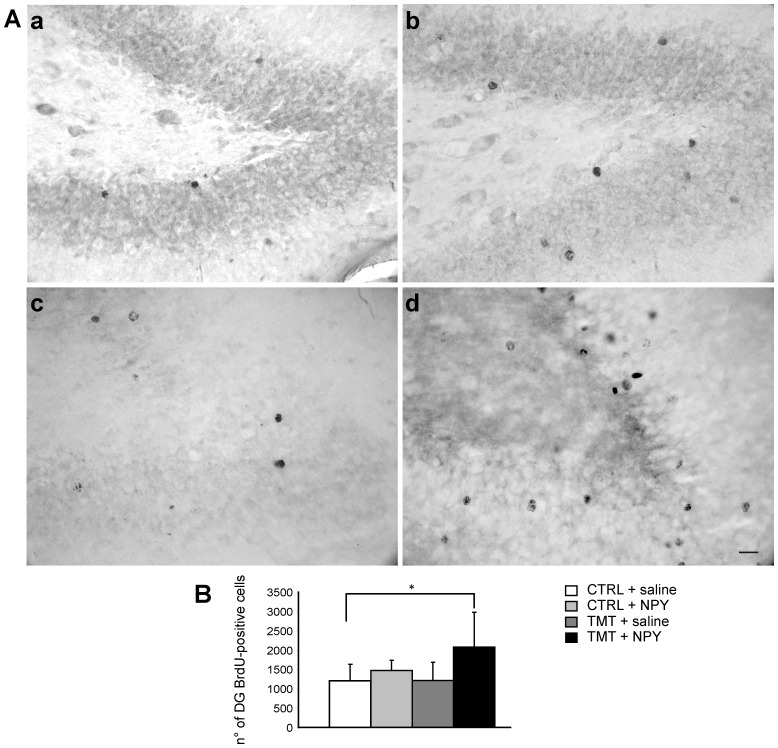
BrdU-immunoreactivity in the DG of the different experimental groups. **A**. Representative micrographs showing DAB-stained BrdU-positive cells in the DG of CTRL+saline- (a), CTRL+NPY- (b), TMT+saline- (c), TMT+NPY (d) -treated rats: an increased number of BrdU-positive newly-generated cells is evident in the DG of TMT+NPY-treated animals (d), when compared with the other groups (a, b, c). Scale bar: 40 µm. **B**. Bar graphs showing the number of BrdU-stained nuclei in the DG of the different experimental groups. A significantly higher number of BrdU-positive cells is evident in TMT+NPY-treated animals compared with the CTRL+saline group. The values are given as means ± SD (*p<0.05).

Light microscopy analysis of BrdU-labelled sections revealed the presence of round and regular BrdU-positive nuclei in the DG of all experimental groups (Fig. 2 Aa–d), being more numerous in the TMT+NPY-treated animals (Fig. 2 Ad).

Unbiased quantitative analysis of DAB-stained BrdU-positive cells, performed to determine whether NPY-induced enhancement of dentate neurogenesis in TMT-treated rats is still evident 30 days after treatment, showed a significant effect of both TMT and NPY treatments (Two-way ANOVA, F_1,24_ = 4.9 for saline vs. NPY, p<0.05, F_1,24_ = 4.8 for CTRL vs. TMT p<0.05); in particular *post hoc* comparison indicated that the number of newly-generated cells was significantly higher in the GL of TMT+NPY-treated rats than in the CTRL+saline group (Bonferroni *post hoc*: p<0.01). No significant statistical difference was evidenced between TMT+NPY and TMT+saline-treated rats (Two-way ANOVA F_1,24_ = 4.9, Bonferroni *post hoc* p>0.05), TMT+saline-treated animals and both CTRL groups (Two-way ANOVA F_1,24_ = 4.8; Bonferroni *post hoc* p>0.05) or between the CTRL+saline and CTRL+NPY groups (two-way ANOVA F_1,24_ = 4.9, Bonferroni *post hoc* p>0.05) (Fig. 2 B).

**Figure 3:**
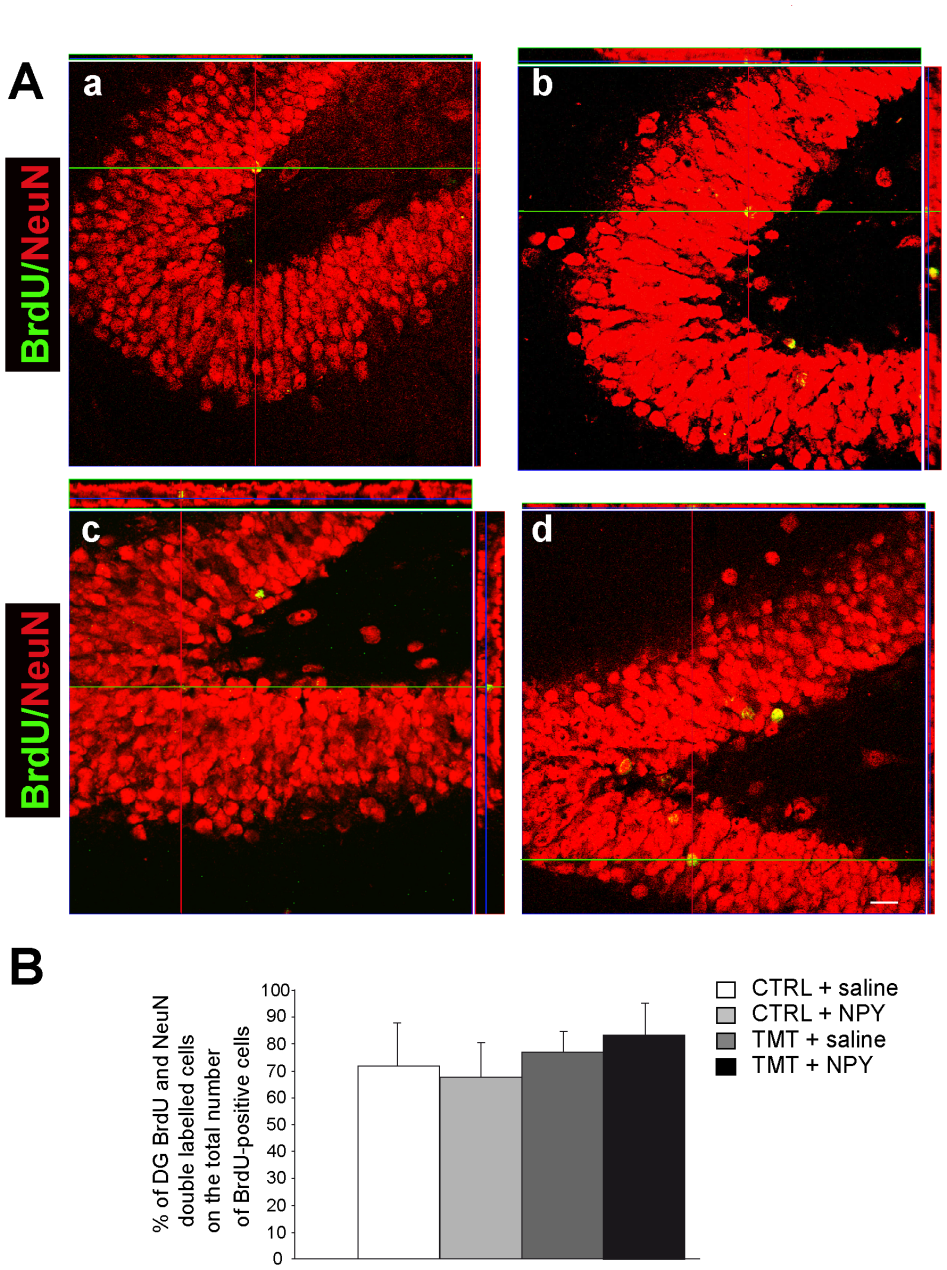
BrdU/NeuN double-stained cells in the DG of the different experimental groups. **A**. Representative confocal microscopy micrographs of CTRL+saline- (a), CTRL+NPY- (b), TMT+saline- (c), TMT+NPY (d) -treated rat DG sections double-labelled for NeuN (red) and BrdU (green): a higher number of BrdU/NeuN-positive cells is evident in TMT+NPY-treated rats (d). Scale bar: 60 µm. **B**. The percentage of BrdU/NeuN double-stained cells is unchanged in the various experimental groups. The values are given as means ± S.D. (*p<0.05).

To determine how many of the newly-generated cells within the DG differentiate into neurons, we performed confocal microscopy analysis of double-stained BrdU-positive cells co-expressing the mature neuronal marker NeuN. More numerous BrdU/NeuN-positive cells were detectable in TMT+NPY-treated rats (Fig. 3 Ad). Quantitative analysis of BrdU/NeuN double-labelled cells indicated that, while a significantly higher number of double-stained cells was evidenced in the TMT+NPY treated group compared with all other groups (see Table S2), the percentage of BrdU/NeuN double-stained cells in relation to the total number of BrdU-positive cells was unchanged among the various experimental groups (two-way ANOVA F_1,8_ = 1,6 p>0.05 for TMT vs. CTRL; two-way ANOVA F_1,8_ = 0,15 p>0.05 for NPY vs. saline) (Fig. 3 B).

It is known that, in the time window ranging from 30 to 45 days after mitosis, newly-generated neurons exhibit peculiar features, including low sensitivity to GABAergic inhibition that distinguish them from mature granule neurons in the DG [53], [57]–[59]. To assess the functional integration of newly-born neurons into the DG network, we studied LTP at the MPP-DGC synapses in slices from rats belonging to the four experimental groups perfused with normal aCSF, i.e., without GABA_A_ receptor antagonists (aCSF-LTP). aCSF-LTP is fairly weak compared to that usually recorded in the presence of GABA_A_ receptor blockers, but the potentiation that is observed can be attributed specifically to the newly-generated neurons [60], [61].

**Figure 4:**
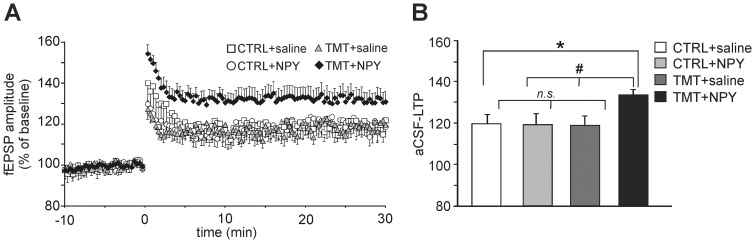
Long-term potentiation (LTP) at medial perforant pathway (MPP)-dentate granule cell (DGC) synapses. **A**. Time course of MPP-DGC LTP induced by high frequency stimulation (HFS) (delivered at time 0) in hippocampal slices from rats belonging to the four experimental groups (CTRL+saline, CTRL+NPY, TMT+saline, TMT+NPY). Recordings were performed in normal aCSF (i.e, in the absence of GABA_A_ receptor blockade) and show enhanced LTP (aCSF-LTP) in slices from the TMT+NPY group compared to all the other experimental groups. Results are expressed as percentages of baseline fEPSP amplitude (100%). **B**. Bar graphs comparing LTP magnitudes observed during the last 5 min of recording in the different experimental groups. Error bars indicate S.E.M. values. *p<0.05 vs CTRL+saline-treated rats; #p<0.05 vs TMT+NPY group, (Student’s t test); n.s., not significant.

In hippocampal slices from CTRL+saline rats fEPSP amplitudes measured 30 min after HFS stimulation were only 119.9±4.2% of baseline values (n = 10 slices from 5 animals; Fig. 4). Comparison with the other experimental groups revealed that aCSF-LTP values were significantly higher in the TMT+NPY-treated group (132.0±4.1%; n = 13 slices from 5 animals) than in all the other groups (TMT+saline: 118.9±4.3%, n = 10 slices from 5 animals; CTRL+NPY: 119.4±5.0%, n = 10 slices from 5 animals; TMT+NPY vs. TMT+saline or CTRL+NPY or CTRL+saline p<0.05), while no significant differences were detectable when comparing TMT+saline vs. CTRL+NPY or CTRL saline (p>0.05; Fig. 4 B), and CTRL+NPY vs. CTRL+saline (p>0.05, Fig. 4 B).

In order to analyse molecular correlates of the maturation and integration of newly-born granular cells in the DG network we evaluated the expression of the transcription factor Klf9, essential for late-phase neuronal maturation during adult hippocampal neurogenesis [62], 30 days after NPY administration. Expression of neural-specific kinase Cdk5, known to play a key role in synapse formation and neuronal maturation during adult neurogenesis [63], was also evaluated at the same time point.

**Figure 5:**
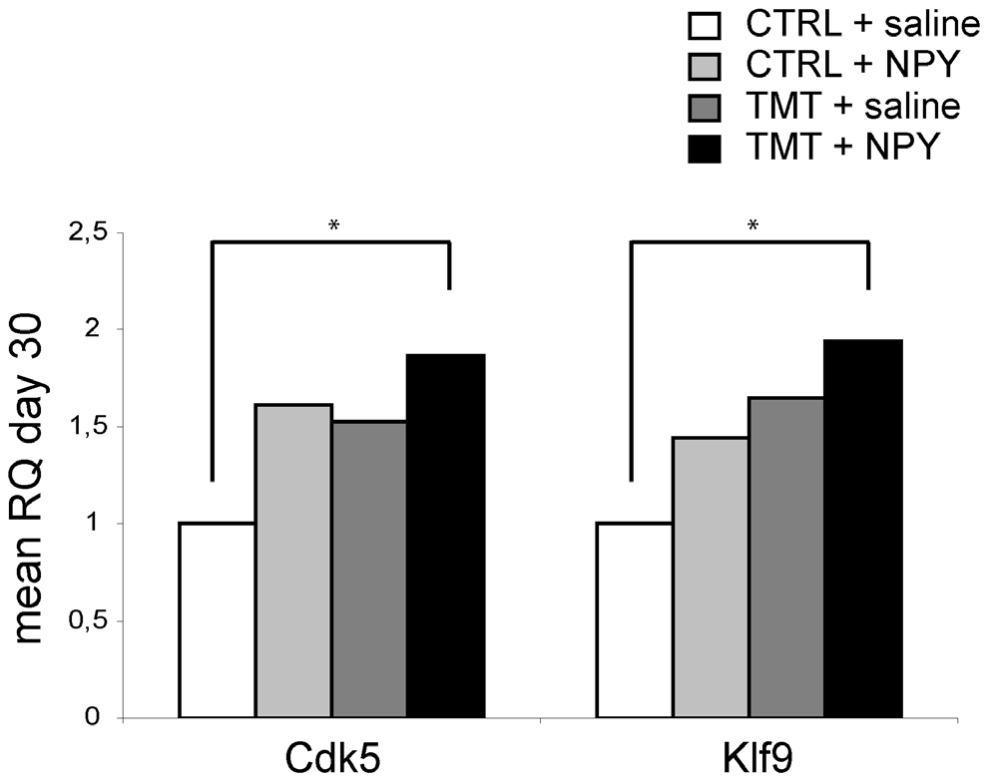
mRNA expression levels of Klf9 and Cdk5 in rat hippocampus in the different experimental groups. Results of quantitative real time-PCR obtained using the ΔΔCt method for calculation of the relative quantity (RQ) of Cdk5 and Klf9 genes tested 30 days after NPY administration. *p<0.05, calculated on mean ΔCt across biological replicates.

qPCR revealed a significant up-regulation of the Klf9 gene in TMT+NPY-treated rats compared with CTRL+saline (p<0.05) (Fig. 5; Table S3). A slight and barely significant upregulation (p = 0.05) was also observed in rats treated with NPY alone (CTRL+NPY) at the same time point (Fig. 5). Similarly, an increase in Cdk5 gene expression was evident in the TMT+NPY group compared with CTRL+saline (p<0.05) (Fig. 5; Table S3).

### Early Molecular Events Activated by NPY Administration Involve Sonic Hedgehog Signalling Pathway Modulation

To investigate the early molecular events mediating NPY-induced neurogenic effects, we evaluated the expression of Shh and its downstream genes, Ptch1, Kif3a and Ccnd1, in the early phases following NPY administration, namely 1, 3 and 5 days after neuropeptide treatment. It is well known that the canonical Shh signalling pathway includes its binding to the Ptch1 receptor, which relieves the Ptch-mediated inhibition of the Smo receptor, which in turn induces a recruitment of Gli transcription factors, resulting in increased expression of Ccnd1 [64]. Genes encoding the intraflagellar transport machinery (including Kif3a) are also required for Shh signalling [65].

**Figure 6:**
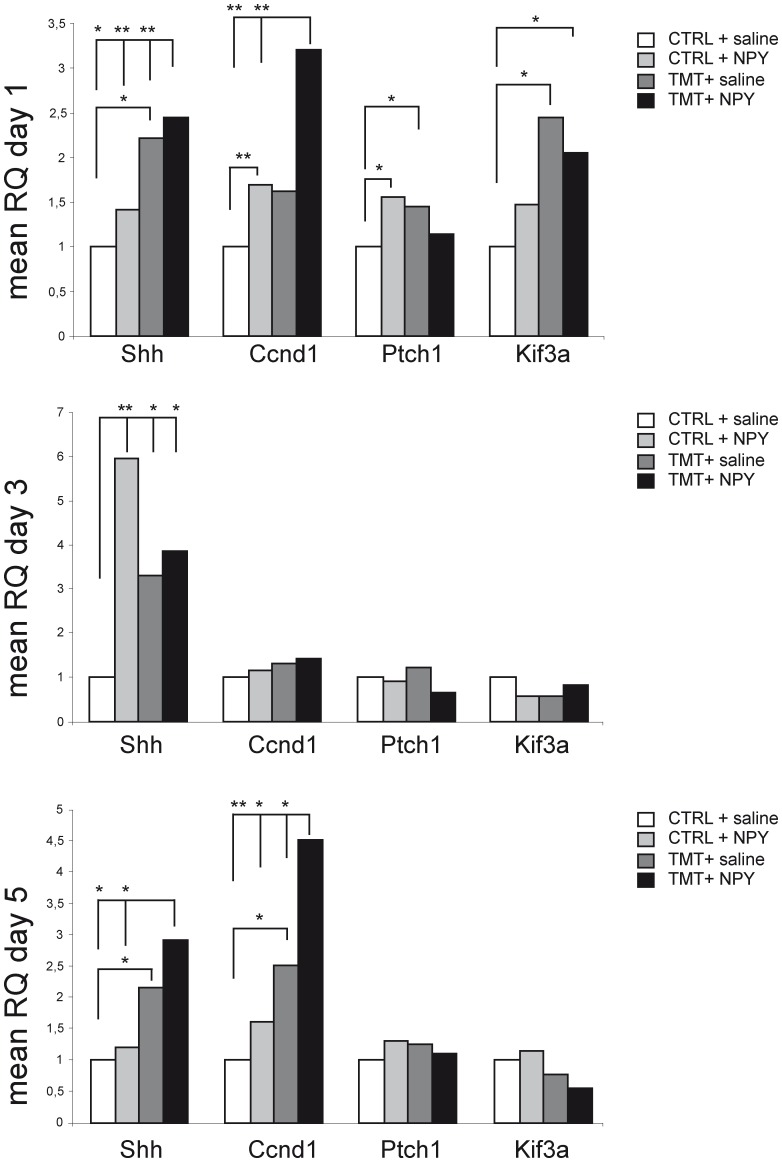
mRNA expression levels of genes related to the Shh pathway in rat hippocampus in the different experimental groups. Results of quantitative real time-PCR obtained using the ΔΔCt method for the calculation of relative quantity (RQ) of Shh, Ccnd1, Ptch1 and Kif3a genes at the three tested time points. *p<0.05, **p<0.001, calculated on mean ΔCt across biological replicates.

One day after NPY administration a statistically significant increase in Shh expression was observed in the TMT+NPY group, when compared with TMT+saline and both CTRL groups (TMT+NPY vs. TMT+saline p<0.001; TMT+NPY vs. CTRL+saline p<0.05; TMT+NPY vs CTRL+NPY p<0.001), accompanied by a significantly increased expression of two Shh downstream genes, namely Kif3a (TMT+NPY vs. CTRL+saline p<0.05) and Ccnd1 (TMT+NPY vs. CTRL+saline or CTRL NPY p<0.001) (Fig. 6, Table S3). At the same time point a significant upregulation of Shh expression was also detectable in the TMT+saline group when compared with CTRL+saline (p<0.05), associated with the concomitant upregulation of Ptch1 and Kif3a (TMT+saline vs CTRL+saline p<0.05) (Fig. 6, Table S3).

Shh expression did not show any significant change in CTRL+NPY-treated animals (CTRL+NPY vs CTRL+saline p>0.05) at this time point, though a significant upregulation of downstream genes Ptch1 (p<0.05) and Ccnd1 (p<0.001) was detectable when compared with CTRL+saline, suggesting that a modulation of the Shh signalling pathway also occurred in this experimental group (Fig. 6, Table S3).

Three days after NPY administration, Shh mRNA levels were still higher in the TMT+NPY and TMT+saline groups, when compared with CTRL+saline (p<0.05). At this time point a significant transient increase in Shh expression was also observed in the CTRL+NPY group compared with CTRL+saline (p<0.001). No significant variation of Ptch1, Kif3a and Ccnd1 expression was detectable among the different experimental groups (p>0.05) (Fig. 6, Table S3).

Five days after NPY administration Shh expression was still significantly upregulated in the TMT+NPY group, when compared with both CTRL groups (TMT+NPY vs CTRL+saline or CTRL+NPY p<0.05), and in TMT+saline, when compared with CTRL+saline (p<0.05). A significant upregulation of Ccnd1 was detectable in both TMT-treated groups, with the highest expression level observed in the TMT+NPY group (TMT+NPY vs TMT+saline p<0.05; TMT+NPY vs CTRL+saline p<0.001; TMT+NPY vs CTRL+NPY p<0.05; TMT+saline vs CTRL+saline p<0.05). No modulation of Shh downstream-related genes (Ptch1 and Kif3a) was detectable at this time point (p>0.05). (Fig. 6, Table S3).

The expression levels of Npy1R, Npy2R, Npy5R were evaluated in the four experimental groups 1 and 3 days after NPY administration. A scrambled-NPY peptide-injected group was also used as control. As expected, the expression pattern of NpyRs indicated an up-regulation of Npy1R, Npy2R, Npy5R in the NPY-treated groups (CTRL+NPY and TMT+NPY) when compared with CTRL+saline group (p<0.05), while no significant modulation was detectable in the scrambled-peptide- and saline-treated animals (p>0.05). This was consistent with our previous results [34] (Fig. S1).

## Discussion

Although the pro-neurogenic role of exogenous NPY has been extensively evaluated in both *in vitro* [11], [66] and *in vivo* experimental models of neurologic disorders [12], [34], evidence that the newly-generated cells survive and become functionally integrated neurons has been poorly pursued. To address this issue, in the present study the long-term *in vivo* effects and potential functional impact of NPY administration in the TMT-model of neurodegeneration and TLE were explored at the molecular, morphological and electrophysiological level.

In line with our previous experiments [34], we found that the proliferative effect exerted on the hippocampal neurogenic niche by a single i.c.v. administration of NPY leads to the production of a significantly higher number of BrdU-positive newly-generated neurons in TMT-treated animals, which is still evident 30 days after treatment.

In this regard the relations among the various experimental groups appear to be changed, when compared with our previous study [34]. This is not surprising, bearing in mind the different BrdU administration schedule, the different method used for quantitative analysis, as well as cell death and/or dilution of label [67] possibly occurring during the longer temporal frame investigated in this study.

No difference in the percentage of BrdU/NeuN double-labelled neurons was detected among the different experimental groups, thus indicating that the pool of newly-generated cells shows similar features in terms of fate determination.

Our electrophysiological data indicate that TMT+NPY-treated animals showed enhanced LTP which correlates with the increased number of newly-born neurons integrated within the DG. Recent studies have demonstrated that the specific contribution of newly-generated cells to LTP at the MPP-DGC synapses can be unravelled performing experiments without blocking GABA_A_ receptors. Differently from more mature DG neurons, the newly-generated cells integrated in the DG 30–45 days after mitosis are indeed insensitive to GABAergic inhibition. In rodents aCSF-LTP is absent when neurogenesis is chemically, physically or genetically reduced, whereas it is enhanced when neurogenesis is increased [53], [59], [61], [68], [69]. We found that aCSF-LTP was similar in all groups except for the TMT+NPY group, in which it was significantly enhanced, thus supporting the functional integration of newly-generated granule cells in the DG network of TMT+NPY-treated animals.

Interestingly, in support of our morphological and physiological results, we observed, in TMT+NPY-treated animals, a concomitant and significant up-regulation of the transcription factor Klf9, considered a specific regulator of late-phase maturation of the dentate granule neurons, being essential for their normal integration into the local circuit [62]. Further support to our conclusions is also given by the increased expression of Cdk5, known to be involved not only in neuronal migration phenomena, but also in dendritic pathfinding, spine formation and neuronal maturation in the newborn cells of adult brain [63], [70], [71].

In order to provide elements useful to understand the molecular cascade triggered by NPY administration and mediating the early phases of its neurogenic effect during the TMT-induced neurodegenerative process, we explored the expression pattern of the Shh pathway, which is crucially required to modulate neural precursor behaviour in the adult DG [35], [40], where it acts as a potent mitogen on the neural progenitor cells of the hippocampal niche [37].

The significantly higher levels of Shh mRNA found in the TMT+NPY-treated group compared with TMT+saline treated rats, along with the modulation of the downstream genes Ptch1 and Ccnd1 detected in CTRL+NPY-treated animals, may suggest a direct role of NPY on Shh pathway modulation. This molecular profile appeared to be especially marked at the earlier time point explored (1 day), though the higher expression of Ccnd1 observed in the TMT+NPY group 5 days after NPY administration may also suggest a persistent effect. These findings are in line with the proliferative effect known to be exerted by Shh on hippocampal progenitors [36], [37], thus further supporting its role in the establishment and maintenance of the hippocampal niche [40]. Notably, the expression of Shh has been suggested to depend on the expression of Sox2, a transcription factor required for the maintenance of progenitor cells in the hippocampus [72], which, according to our previous data, is significantly upregulated by NPY administration in TMT-treated rats at the same time point [34].

Our novel findings of a prolonged increase in Shh expression in TMT+saline-treated groups is in line with previous evidence suggesting a relationship between Shh expression and TLE in both experimental models and human epilepsy [73], [74]. It has been hypothesised, in this regard, that the Shh pathway may be an important mechanism through which epilepsy enhances adult hippocampal neurogenesis [73]. Given that this pathway has been proposed to exert neuroprotective effects in many neurologic diseases [40], [75], [76], we may also hypothesise that the enhanced Shh expression during TMT-induced neurodegeneration could be part of the early activation of repair mechanisms in the injured hippocampus, albeit with no reparative outcome. On the other hand, a role in the development of neuronal death and TLE cannot be excluded, as suggested by other groups [74].

More in general, in line with our previous findings, the described effects of NPY administration are particularly evident in the TMT-treated animals. Many studies have demonstrated that several factors that are known to promote neural precursor proliferation and survival (including nerve growth factor, brain-derived neurotrophic factor, fibroblast growth factor-2, vascular endothelial growth factor, and also Shh) are upregulated in the hippocampus after acute seizures [77]. Thus we may speculate that micro-environmental changes induced by epilepsy and/or neurodegeneration could strengthen the NPY-mediated influence on the hippocampal niche, thus promoting significant neurogenic outcomes.

The present study provides novel evidence that a single NPY administration during TMT-induced neurodegeneration triggers early activation of the Shh pathway and exerts long-term effects on dentate neurogenesis, resulting in the production of a population of functionally integrated granule cells. It is well known that this population of adult-born neurons plays a key role in learning and memory processes [78], [79], thus contributing to the physiological functions of the hippocampus. Taking into account the relevance of hippocampal dysfunction in TLE and neurodegeneration, our observations offer information that is potentially useful in the light of possible restorative outcomes.

## Supporting Information

Figure S1 **mRNA expression levels of NPY receptor genes after NPY-, scrambled-NPY- or saline administration in TMT-treated and CTRL rats.** **A.** The histogram shows the results of quantitative real time-PCR obtained using the ΔΔCt method for calculation of the relative quantity (RQ) of NPY receptor genes (NPY1R, NPY2R, NPY5R) tested 1 and 3 days after NPY-, scrambled NPY- or saline administration in TMT-treated and CTRL rat hippocampi. *p<0.05, calculated on mean ΔCt across biological replicates. **B.** The table shows mean ΔCt, SD, SEM and p values referred to qPCR results.
(TIF)

Table S1 **Oligonucleotide primer sequences.** The table indicates the oligonucleotide primer sequences used in qPCR analysis.
(DOC)

Table S2 **Cell count results of double-stained BrdU/NeuN positive cells.** The table indicates the values of the total numbers of BrdU/NeuN double-stained cells ± SD in the DG granular layer of the different experimental groups. Statistical analysis showed that the number of BrdU/NeuN double-labelled cells was significantly higher in TMT+NPY-treated rats compared with both CTRL groups (Two-way ANOVA, F_1,8_ = 32.5 p<0.001) and TMT+saline group (Two-way ANOVA, F_1,8_ = 5.37 p<0.05).
(DOC)

Table S3 **Mean ΔCt values, SD, SEM and p values referred to qPCR analysis.** Table S3 shows the values of mean ΔCt, SD, SEM and p referred to qPCR analysis of Shh, Ptch1, Ccnd1, Kif3a gene expression performed 1, 3 and 5 days after NPY treatment (A, B, C) and to Klf9 and Cdk5 gene expression performed 30 days after NPY treatment (D).
(DOC)
