# Supplementary figures and images for: The Neurogenic Effects of Exogenous Neuropeptide Y: Early Molecular Events and Long-Lasting Effects in the Hippocampus of Trimethyltin-Treated Rats
Source: PLoS One. 2014 Feb 7;9(2):e88294. doi: 10.1371/journal.pone.0088294 (PMC3917853; doi:10.1371/journal.pone.0088294)

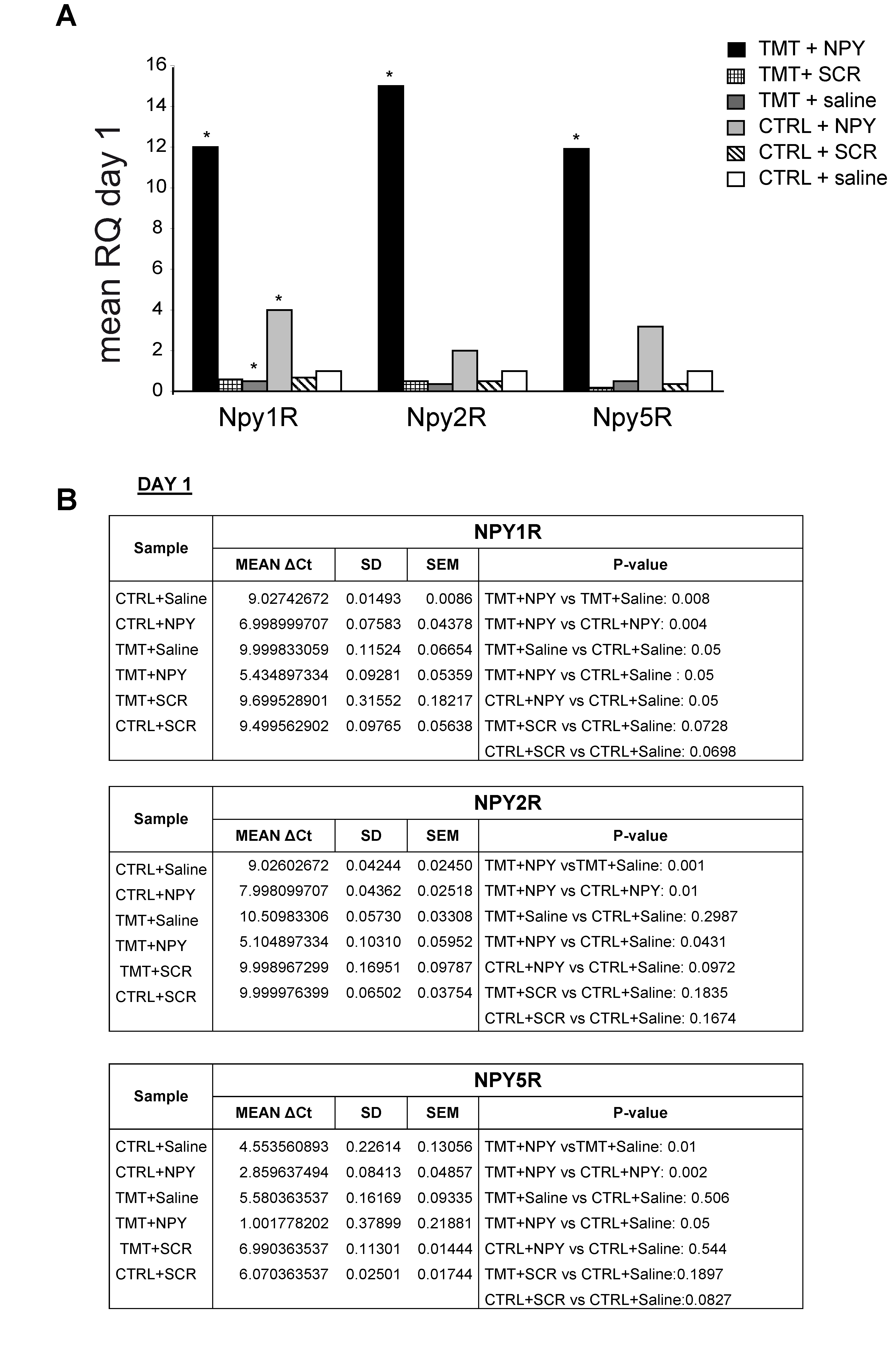

Supplement: Figure S1 — mRNA expression levels of NPY receptor genes after NPY-, scrambled-NPY- or saline administration in TMT-treated and CTRL rats. A. The histogram shows the results of quantitative real time-PCR obtained using the ΔΔCt method for calculation of the relative quantity (RQ) of NPY receptor genes (NPY1R, NPY2R, NPY5R) tested 1 and 3 days after NPY-, scrambled NPY- or saline administration in TMT-treated and CTRL rat hippocampi. *p<0.05, calculated on mean ΔCt across biological replicates. B. The table shows mean ΔCt, SD, SEM and p values referred to qPCR results. (TIF) [file pone.0088294.s001.tif]
