# Supplementary material for: The Neurogenic Effects of Exogenous Neuropeptide Y: Early Molecular Events and Long-Lasting Effects in the Hippocampus of Trimethyltin-Treated Rats
Source: PLoS One. 2014 Feb 7;9(2):e88294. doi: 10.1371/journal.pone.0088294 (PMC3917853; doi:10.1371/journal.pone.0088294)
Supplement: Table S1 — Oligonucleotide primer sequences. The table indicates the oligonucleotide primer sequences used in qPCR analysis. (DOC) [file pone.0088294.s002.doc]

| **Gene Symbol** | **Forward primer** | **Reverse primer** |
| --- | --- | --- |
| **Shh** | 5’- acggcaccattctcatcaac -3’ | 5’- ctcctcgatgactgcgtagc -3’ |
| **Ptch1** | 5’- aggcactatgaagcacagca -3’ | 5’- ggcttccacaatcacttggt -3’ |
| **Ccnd1** | 5’- cagattgaagcccttctgga -3’ | 5’- cttgggatcgatgttctgct -3’ |
| **Kif3a** | 5’- tggctgcaaagtcagagatg -3’ | 5’- ttccctctgatgctcttgct -3’ |
| **Klf9** | 5’- aatctgggtcgagtccttcc -3’ | 5’- tgccagaatcctgtctctcc -3’ |
| **Cdk5** | 5’- gcaatgatgtggatgaccag -3’ | 5’- ggtgtccctagcagtcgaaa -3’ |
| **Npy1r** | 5’- tcgagacgacgactacgaga-3’ | 5’- cacgtccgtatgcatggtag-3’ |
| **Npy2r** | 5’- tgcattgtctaccacctgga-3’ | 5’- aggaagctgatttgcttgga-3’ |
| **Npy5r** | 5’- atacagctgctgctcggaat-3’ | 5’- acgctgcctctgtagtcctc-3’ |
