# Supplementary material for: The Neurogenic Effects of Exogenous Neuropeptide Y: Early Molecular Events and Long-Lasting Effects in the Hippocampus of Trimethyltin-Treated Rats
Source: PLoS One. 2014 Feb 7;9(2):e88294. doi: 10.1371/journal.pone.0088294 (PMC3917853; doi:10.1371/journal.pone.0088294)
Supplement: Table S2 — Cell count results of double-stained BrdU/NeuN positive cells. The table indicates the values of the total numbers of BrdU/NeuN double-stained cells ± SD in the DG granular layer of the different experimental groups. Statistical analysis showed that the number of BrdU/NeuN double-labelled cells was significantly higher in TMT+NPY-treated rats compared with both CTRL groups (Two-way ANOVA, F1,8 = 32.5 p<0.001) and TMT+saline group (Two-way ANOVA, F1,8 = 5.37 p<0.05). (DOC) [file pone.0088294.s003.doc]

|  | **CTRL+saline** | **CTRL+NPY** | **TMT+saline** | **TMT+NPY** |
| --- | --- | --- | --- | --- |
| **BrdU /NeuN positive-cells** | 253.867±145,7 | 201.600± 73.2 | 476.000±101.2 | 903.467± 204.6 |
