# Supplementary material for: The Neurogenic Effects of Exogenous Neuropeptide Y: Early Molecular Events and Long-Lasting Effects in the Hippocampus of Trimethyltin-Treated Rats
Source: PLoS One. 2014 Feb 7;9(2):e88294. doi: 10.1371/journal.pone.0088294 (PMC3917853; doi:10.1371/journal.pone.0088294)
Supplement: Table S3 — Mean ΔCt values, SD, SEM and p values referred to qPCR analysis. Table S3 shows the values of mean ΔCt, SD, SEM and p referred to qPCR analysis of Shh, Ptch1, Ccnd1, Kif3a gene expression performed 1, 3 and 5 days after NPY treatment (A, B, C) and to Klf9 and Cdk5 gene expression performed 30 days after NPY treatment (D). (DOC) [file pone.0088294.s004.doc]

A - DAY 1

| **Sample** | **Shh** | | | | | |
| --- | --- | --- | --- | --- | --- | --- |
|  | **MEAN ΔCt** | | **SD** | **SEM** | | **p-value** |
| CTRL+Saline | 14.57973782 | | 0.58837 | 0.33969 | | TMT+NPY vs TMT+Saline: 0.0008 |
| CTRL+NPY | 14.0834744 | | 0.11450 | 0.06611 | | TMT+NPY vs CTRL+NPY 0.0003 |
| TMT+Saline | 13.43424699 | | 0.01400 | 0.00808 | | TMT+Saline vs CTRL+Saline: 0.02 |
| TMT+NPY | 13.29343947 | | 0.02300 | 0.01328 | | TMT+NPY vs CTRL+Saline : 0.0194 |
|  |  | |  |  | | CTRL+NPY vs CTRL+Saline: 0.2328 |
|  |  | |  |  | |  |
| **Sample** | **Ptch1** | | | | | |
|  | **MEAN ΔCt** | **SD** | | **SEM** | **p-value** | |
| CTRL+Saline | 8.84123373 | 0.30885 | | 0.17832 | TMT+NPY vs TMT+Saline: 0.1376 | |
| CTRL+NPY | 8.19797586 | 0.02750 | | 0.01588 | TMT+NPY vs CTRL+NPY: 0.07 | |
| TMT+Saline | 8.295488582 | 0.01200 | | 0.00693 | TMT+Saline vs CTRL+Saline: 0.037 | |
| TMT+NPY | 8.649287692 | 0.012113 | | 0.19081 | TMT+NPY vs CTRL+Saline: 0.503 | |
|  |  |  | |  | CTRL+NPY vs CTRL+Saline: 0.012 | |
|  |  |  | |  |  | |
| **Sample** | **Ccnd1** | | | | | |
|  | **MEAN ΔCt** | **SD** | | **SEM** | **p-value** | |
| CTRL+Saline | 10.5860397 | 0.08708 | | 0.05027 | TMT+NPY vs TMT+Saline: 0.059 | |
| CTRL+NPY | 9.820602184 | 0.12000 | | 0.06928 | TMT+NPY vs CTRL+NPY: 0.0019 | |
| TMT+Saline | 9.874382496 | 0.61800 | | 0.35680 | TMT+Saline vs CTRL+Saline: 0.1195 | |
| TMT+NPY | 8.90483141 | 0.18385 | | 0.10614 | TMT+NPY vs CTRL+Saline: 0.0001 | |
|  |  |  | |  | CTRL+NPY vs CTRL+Saline: 0.0009 | |
|  |  |  | |  |  | |
| **Sample** | **Kif3a** | | | | | |
|  | **MEAN ΔCt** | **SD** | | **SEM** | **p-value** | |
| CTRL+Saline | 7.704076038 | 0.37350 | | 0.21564 | TMT+NPY vs TMT+Saline: 0.0934 | |
| CTRL+NPY | 7.148339995 | 0.51300 | | 0.29618 | TMT+NPY vs CTRL+NPY: 0.191 | |
| TMT+Saline | 6.410698825 | 0.07100 | | 0.04099 | TMT+Saline vs CTRL+Saline: 0.004 | |
| TMT+NPY | 6.654984312 | 0.17950 | | 0.10363 | TMT+NPY vs CTRL+Saline: 0.011 | |
|  |  |  | |  | CTRL+NPY vs CTRL+Saline: 0.2039 | |

B - DAY 3

| **Sample** | **Shh** | | | |
| --- | --- | --- | --- | --- |
|  | **MEAN ΔCt** | **SD** | **SEM** | **p-value** |
| CTRL+Saline | 7.905126187 | 0.8035 | 0.46390 | TMT+NPY vs TMT+Saline: 0.1103 |
| CTRL+NPY | 5.338434123 | 0.449 | 0.25923 | TMT+NPY vs CTRL+NPY 0.079 |
| TMT+Saline | 6.181983136 | 0.1365 | 0.07881 | TMT+Saline vs CTRL+Saline:0.021 |
| TMT+NPY | 5.967003422 | 0.122432 | 0.05957 | TMT+NPY vs CTRL+Saline : 0.014 |
|  |  |  |  | CTRL+NPY vs CTRL+Saline: 0.0085 |
|  |  |  |  |  |
| **Sample** | **Ptch1** | | | |
|  | **MEAN ΔCt** | **SD** | **SEM** | **p-value** |
| CTRL+Saline | 5.615111669 | 0.30885 | 0.27181 | TMT+NPY vs TMT+Saline: 0.08 |
| CTRL+NPY | 5.710231622 | 0.02750 | 0.26082 | TMT+NPY vs CTRL+NPY: 0.1187 |
| TMT+Saline | 5.438976606 | 0.01200 | 0.06569 | TMT+Saline vs CTRL+Saline: 0.5625 |
| TMT+NPY | 6.328181903 | 0.012113 | 0.16902 | TMT+NPY vs CTRL+Saline: 0.08 |
|  |  |  |  | CTRL+NPY vs CTRL+Saline: 0.8127 |
|  |  |  |  |  |
| **Sample** | **Ccnd1** | | | |
|  | **MEAN ΔCt** | **SD** | **SEM** | **p-value** |
| CTRL+Saline | 5.802971235 | 0.11150 | 0.06437 | TMT+NPY vs TMT+Saline: 0.9715 |
| CTRL+NPY | 5.426093954 | 0.28104 | 0.16226 | TMT+NPY vs CTRL+NPY: 0.8578 |
| TMT+Saline | 5.339780211 | 0.32035 | 0.18495 | TMT+Saline vs CTRL+Saline: 0.0773 |
| TMT+NPY | 5.354464537 | 0.58600 | 0.33833 | TMT+NPY vs CTRL+Saline: 0.2626 |
|  |  |  |  | CTRL+NPY vs CTRL+Saline: 0.0969 |
|  |  |  |  |  |
| **Sample** | **Kif3a** | | | |
|  | **MEAN ΔCt** | **SD** | **SEM** | **p-value** |
| CTRL+Saline | 3.19510142 | 0.40450 | 0.23354 | TMT+NPY vs TMT+Saline: 0.1174 |
| CTRL+NPY | 3.984916135 | 1.07049 | 0.61805 | TMT+NPY vs CTRL+NPY: 0.4874 |
| TMT+Saline | 4.028608958 | 0.43454 | 0.25088 | TMT+Saline vs CTRL+Saline: 0.07 |
| TMT+NPY | 3.509620508 | 0.12301 | 0.07102 | TMT+NPY vs CTRL+Saline: 0.2673 |
|  |  |  |  | CTRL+NPY vs CTRL+Saline: 0.298 |

C - DAY 5

| **Sample** | **Shh** | | | |
| --- | --- | --- | --- | --- |
|  | **MEAN ΔCt** | **SD** | **SEM** | **p-value** |
| CTRL+Saline | 11.95253976 | 0.54589 | 0.31517 | TMT+NPY vs TMT+Saline: 0.2854 |
| CTRL+NPY | 11.69298459 | 0.18481 | 0.10670 | TMT+NPY vs CTRL+NPY 0.02 |
| TMT+Saline | 10.84766293 | 0.11243 | 0.06491 | TMT+Saline vs CTRL+Saline:0.02 |
| TMT+NPY | 10.41223462 | 0.60260 | 0.34791 | TMT+NPY vs CTRL+Saline : 0.03 |
|  |  |  |  | CTRL+NPY vs CTRL+Saline: 0.4793 |
|  |  |  |  |  |
| **Sample** | **Ptch1** | | | |
|  | **MEAN ΔCt** | **SD** | **SEM** | **p-value** |
| CTRL+Saline | 7.814630507 | 0.61602 | 0.35566 | TMT+NPY vs TMT+Saline: 0.4536 |
| CTRL+NPY | 7.426028253 | 0.22234 | 0.12837 | TMT+NPY vs CTRL+NPY: 0.1404 |
| TMT+Saline | 7.515069008 | 0.32522 | 0.18776 | TMT+Saline vs CTRL+Saline: 0.5225 |
| TMT+NPY | 7.67530473 | 0.07711 | 0.04452 | TMT+NPY vs CTRL+Saline: 0.7477 |
|  |  |  |  | CTRL+NPY vs CTRL+Saline: 0.3874 |
|  |  |  |  |  |
| **Sample** | **Ccnd1** | | | |
|  | **MEAN ΔCt** | **SD** | **SEM** | **p-value** |
| CTRL+Saline | 8.884599689 | 0.44400 | 0.25634 | TMT+NPY vs TMT+Saline: 0.0367 |
| CTRL+NPY | 8.195441883 | 0.35881 | 0.20716 | TMT+NPY vs CTRL+NPY: 0.0003 |
| TMT+Saline | 7.563882192 | 0.42381 | 0.24469 | TMT+Saline vs CTRL+Saline: 0.0204 |
| TMT+NPY | 6.749426391 | 0.17073 | 0.09857 | TMT+NPY vs CTRL+Saline: 0.001 |
|  |  |  |  | CTRL+NPY vs CTRL+Saline: 0.4879 |
|  |  |  |  |  |
| **Sample** | **Kif3a** | | | |
|  | **MEAN ΔCt** | **SD** | **SEM** | **p-value** |
| CTRL+Saline | 5.519176163 | 0.77691 | 0.44855 | TMT+NPY vs TMT+Saline: 0.3272 |
| CTRL+NPY | 5.335800488 | 0.13901 | 0.08026 | TMT+NPY vs CTRL+NPY: 0.056 |
| TMT+Saline | 5.915610312 | 0.46363 | 0.26768 | TMT+Saline vs CTRL+Saline: 0.4903 |
| TMT+NPY | 6.455735523 | 0.69952 | 0.40387 | TMT+NPY vs CTRL+Saline: 0.1956 |
|  |  |  |  | CTRL+NPY vs CTRL+Saline: 0.708 |
|  |  |  |  |  |

D - DAY 30

| **Sample** | **Klf9** | | | |
| --- | --- | --- | --- | --- |
|  | **MEAN ΔCt** | **SD** | **SEM** | **p-value** |
| CTRL+Saline | 3.447604067 | 0.60472 | 0.34913 | TMT+NPY vs TMT+Saline: 0.5485 |
| CTRL+NPY | 2.924458186 | 0.52475 | 0.30296 | TMT+NPY vs CTRL+NPY 0.2865 |
| TMT+Saline | 2.724175771 | 0.53535 | 0.30908 | TMT+Saline vs CTRL+Saline:0.1956 |
| TMT+NPY | 2.376737714 | 0.16869 | 0.09739 | TMT+NPY vs CTRL+Saline : 0.0279 |
|  |  |  |  | CTRL+NPY vs CTRL+Saline: 0.742 |
|  |  |  |  |  |
| **Sample** | **Cdk5** | | | |
|  | **MEAN ΔCt** | **SD** | **SEM** | **p-value** |
| CTRL+Saline | 5.414402472 | 0.62426 | 0.36042 | TMT+NPY vs TMT+Saline: 0.7854 |
| CTRL+NPY | 4.720440865 | 0.55042 | 0.31779 | TMT+NPY vs CTRL+NPY: 0.5414 |
| TMT+Saline | 4.796539313 | 0.11400 | 0.06582 | TMT+Saline vs CTRL+Saline: 0.0519 |
| TMT+NPY | 4.508317032 | 0.01800 | 0.01039 | TMT+NPY vs CTRL+Saline: 0.005 |
|  |  |  |  | CTRL+NPY vs CTRL+Saline: 0.369 |
